# Comparison of extracellular vesicle isolation processes for therapeutic applications

**DOI:** 10.1177/20417314231174609

**Published:** 2023-05-23

**Authors:** Soraya Williams, Maria Fernandez-Rhodes, Alice Law, Ben Peacock, Mark P. Lewis, Owen G. Davies

**Affiliations:** 1 School of Sport, Exercise and Health Sciences, Loughborough University, Loughborough, UK; 2 NanoFCM Co., Ltd, Medicity, Nottingham, UK

**Keywords:** Extracellular vesicles, isolation methods, ultracentrifugation, size exclusion chromatography, therapeutics

## Abstract

While extracellular vesicles (EVs) continue to gain interest for therapeutic applications, their clinical translation is limited by a lack of optimal isolation methods. We sought to determine how universally applied isolation methods impact EV purity and yield. EVs were isolated by ultracentrifugation (UC), polyethylene glycol precipitation, Total Exosome Isolation Reagent, an aqueous two-phase system with and without repeat washes or size exclusion chromatography (SEC). EV-like particles could be detected for all isolation methods but varied in their purity and relative expression of surface markers (Alix, Annexin A2, CD9, CD63 and CD81). Assessments of sample purity were dependent on the specificity of characterisation method applied, with total particle counts and particle to protein (PtP) ratios often not aligning with quantitative measures of tetraspanin surface markers obtained using high-resolution nano-flow cytometry. While SEC resulted in the isolation of fewer particles with a relatively low PtP ratio (1.12 × 10^7^ ± 1.43 × 10^6^ vs highest recorded; ATPS/R 2.01 × 10^8^ ± 1.15 × 10^9^, *p* ⩽ 0.05), EVs isolated using this method displayed a comparatively high level of tetraspanin positivity (e.g. ExoELISA CD63⁺ particles; 1.36 × 10^11^ *±* 1.18 × 10^10^ vs ATPS/R 2.58 × 10^10^ *±* 1.92 × 10^9^, *p* ⩽ 0.001). Results originating from an accompanying survey designed to evaluate pragmatic considerations surrounding method implementation (e.g. scalability and cost) identified that SEC and UC were favoured for overall efficiency. However, reservations were highlighted in the scalability of these methods, which could potentially hinder downstream therapeutic applications. In conclusion, variations in sample purity and yield were evident between isolation methods, while standard non-specific assessments of sample purity did not align with advanced quantitative high-resolution analysis of EV surface markers. Reproducible and specific assessments of EV purity will be critical for informing therapeutic studies.

## Introduction

Extracellular vesicles (EVs) continue to gain interest for therapeutic applications.^1^ Their ability to package and transfer intrinsically biocompatible molecules in physiological and pathophysiological processes has made them highly appealing for therapeutic and diagnostic applications.^2–4^ Therapeutic applications have been observed in pioneering studies demonstrating the capability of EVs to deliver therapeutic molecules such as siRNAs,^5^ as well as in their application for regenerative processes such as wound healing^6–8^ and immunomodulation.^9–11^ Additionally, the utilisation of EVs allows for the development of cell-free therapies that offer enhanced safety and storage options when compared with cell-based alternatives.^3,12^ While the potential to lyophilise EVs may give rise to off-the-shelf biotherapeutics in the future.^13,14^ However, to date, clinical translation has been limited by the inability to recover high yields of therapeutically potent EVs.^15,16^ As such, there is a need to understand how commonly applied EV isolation methods impact yield and purity.

Currently there is no gold standard EV isolation method, with each presenting advantages and limitations.^17^ Ultracentrifugation (UC) remains the most frequently applied method,^18,19^ separating particles by size and density. It has advantages for therapeutic applications due to its methodological simplicity and cost-efficiency. However, it does require specialist equipment and is known to result in low purity EV preparations.^20^ Other widely adopted methods include the use of precipitation reagents such as polyethylene glycol (PEG). PEG is frequently applied clinically and incorporated in many commercially available nanoparticle formulations such as the chemotherapeutic liposomal medication Doxil^21^ and the recent mRNA-based covid vaccinations.^22^ In the context of EV isolation, PEG precipitation is scalable, cost-effective and does not require specialist equipment. There are a number of drawbacks though including the co-isolation of non-EV proteins (e.g. albumin),^23^ potential immunogenicity^24^ and the reagents interference with down-stream analysis methods such as mass spectrometry.^25,26^ However, when combined with additional clean up steps, such as ultrafiltration (UF) or size-exclusion chromatography (SEC), it offers a potentially translational approach for manufacturing EV therapies. This is perhaps best exemplified by the recent application of this method in a clinical study for the treatment of graft versus host disease.^27,28^ In addition to PEG precipitation, several commercial precipitation reagents are also available, including Total Exosome Isolation Reagent (Invitrogen) and ExoQuick (System Biosciences).^29^ Commercially available kits are readily accessible, do not require specialist training and are relatively high throughput. However, they encounter many of the same disadvantages as PEG precipitation and scalability is dependent upon product cost and its continued availability.^31^ A variation on standard precipitation methods is the aqueous two-phase system (ATPS), currently utilised for virus purification.^30,31^ This method separates EVs by the partitioning of two-phases using PEG and Dextran (DEX), separating proteins into the top PEG-phase and EVs into the bottom DEX phase.^30,32^ Purity can be further increased by replacing the top PEG phase and repeating the process for enhanced removal of proteins.^33^ However, this method has not yet been widely applied for the isolation of EVs and uncertainty lies around the utilisation of DEX in terms of both interference with downstream analyses and clinical safety.^34^ Lastly, an increasingly applied method for isolating EVs is SEC, which isolates EVs into multiple fractions based on size. This can be achieved using both commercially available columns (e.g. Izon science and Cell Guidance Systems)^35,36^ or those manufactured in house (e.g. Sepharose).^37,38^ The process can also be partially automated (e.g. using an automatic fraction collector) to improve reproducibility.^39^ The major advantage of SEC is its ability to fraction co-isolated soluble proteins and lipoproteins from EV populations.^40^ However, this method is only applicable when utilising relatively small sample volumes and therefore is frequently combined with a pre-concentration step (e.g. UF) to enable the processing of therapeutically relevant volumes.

**Figure 1.**
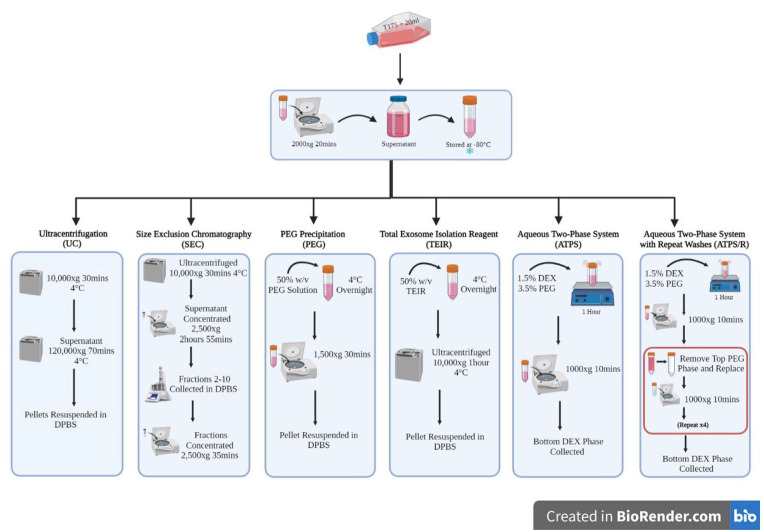
Schematic overview of experimental workflow for EV isolation methods. An overview of conditioned medium processing and downstream EV isolation methods applied in this study. Conditioned medium was centrifuged at 2000 × g for 20-min to remove cells and debris. EV containing fractions were then isolated using UC, SEC, PEG precipitation, commercially available TEIR, ATPS and ATPS/R. All EV preparations were stored at −80℃.

In this study we sought to compare a range of universally applied EV isolation methods (Figure 1: UC, PEG precipitation, TEIR, ATPS and SEC,), to determine their relative impact on EV purity and yield. While more specialised approaches such as tangential flow filtration (TFF) and immunoaffinity isolation are starting to become utilised within the field, they are currently not widely accessible in a standard laboratory setting (TFF) or require in depth knowledge of protein targets on the EV surfaceome (immunoaffinity). As such we chose to focus on widely utilised, readily available and unspecialised methods that can be easily adopted in a standard laboratory setting. The resulting data was combined with a survey that sought to evaluate pragmatic considerations surrounding the implementation of EV isolation protocols (e.g. scalability, accessibility and cost), as this will also be critical to the development of EV therapies.

An overview of conditioned medium processing and downstream EV isolation methods applied in this study. Conditioned medium was centrifuged at 2000 × *g* for 20-min to remove cells and debris. EV containing fractions were then isolated using UC, SEC, PEG precipitation, commercially available TEIR, ATPS and ATPS/R. All EV preparations were stored at −80℃.

## Materials and methods

### C2C12 cell culture and conditioned media collection

Passage 8 C2C12 murine skeletal muscle myoblast cells (ECACC, Sigma-Aldrich) were seeded at a density of 1.5 × 10^6^ in T175 vented culture flasks (Nunclon™ Delta Surface, ThermoFisher Scientific) and incubated in a 5% CO_2_ humidified atmosphere at 37°C. C2C12 cells were selected as a high-throughput immortalised cell source for rapid EV isolation and processing. Cells were cultured until confluent in growth media (GM); composed of high glucose Dulbecco’s Modified Eagle’s Medium (DMEM, Sigma-Aldrich) supplemented with 20% foetal bovine serum (FBS, Pan Biotech) and 1% Penicillin/Streptomycin (P/S, Gibco). Upon reaching confluence, GM was replaced with differentiation media (DM); composed of high glucose DMEM supplemented with 1% P/S and 2% EV-depleted horse serum (HS, HyClone). This provided an EV-depleted low-serum system, minimising residual serum EVs post depletion.^41^ EVs were depleted from HS by ultracentrifugation at 120,000 × *g* for 16-h utilising 20 mL polycarbonate ultracentrifuge tubes (S309156A, SLS) in a Himac CS150FNX ultracentrifuge with S50A rotor. After 5-days in DM the conditioned media was collected and centrifuged at 2000 × *g* for 20-min to remove cells and debris. The supernatant was stored at −80℃.

## EV isolation

### Ultracentrifugation

Conditioned medium was ultracentrifuged at 10,000 × *g* for 30-min at 4℃. The supernatant was then collected and ultracentrifuged at 120,000 × *g* for 70-min at 4℃. All ultracentrifugation steps were performed utilising 20 mL polycarbonate ultracentrifuge tubes (S309156A, SLS) in a Himac CS150FNX ultracentrifuge with S50A rotor. The supernatant was discarded and each pellet re-suspended in DPBS (300 µL per 20 mL conditioned media isolated from, Sigma-Aldrich).

### Size exclusion chromatography

Conditioned medium was ultracentrifuged at 10,000 × *g* for 30-min at 4℃. The resulting supernatant was centrifuged in a Vivaspin^®^20 ultrafiltration unit (ThermoFisher Scientific) at 2500 × *g*, concentrating samples to 500 µL (per 20 mL of conditioned media) before passing through an Izon automated fraction collector (Izon qEV AFC). Fractions 2–10 were combined and re-concentrated at 2500 × *g* to a final volume of 500 µL using a second Vivaspin^®^20 ultrafiltration unit. Fraction and filter selections were optimised in-house.

### Polyethylene glycol precipitation

Polyethylene glycol (PEG) precipitation was performed using PEG MW 6000 (Sigma-Aldrich). PEG solution was made up in DPBS and placed on a shaker until dissolved (within 4-h). The PEG solution was added at a 50% w/v to conditioned medium with a final concentration of 10%, vortexed and incubated overnight at 4°C. The following day the sample was centrifuged at 1500 × *g* for 30-min. The supernatant was discarded and the pellet re-suspended in DPBS.

### Total exosome isolation reagent

Commercially available total exosome isolation reagent (TEIR, Invitrogen) was added at 50% w/v to conditioned medium and incubated overnight at 4°C. The following day the samples were ultracentrifuged at 10,000 × *g* for 1-h at 4°C. The supernatant was discarded and the pellet re-suspended in DPBS.

### Aqueous two-phase system

An aqueous two-phase system (ATPS) was made up by adding 3.5% PEG 35,000 (Sigma-Aldrich) and 1.5% DEX 450,000–650,000 (Sigma-Aldrich) polymers directly in the conditioned medium and placing on a shaker until fully dissolved (1-h) at 4℃. Once dissolved, the solution was vortexed and centrifuged at 1000 × *g* for 10-min. Both the top PEG-phase (approx. 17.5 mL) and interface (1.5 mL to ensure full removal) were discarded and the bottom EV-containing DEX-phase (approx. 1 mL) collected.

### Aqueous two-phase system with repeat washes

The ATPS method was repeated with additional wash steps (ATPS/R) to further purify by enhanced removal of proteins. Upon completion of the standard ATPS protocol, the top PEG-phase was replaced with equal volumes of fresh PEG-phase, without disrupting the interface and bottom EV-containing DEX-phase. The sample was then vortexed and centrifuged at 1000 × *g* for 10-min. This was repeated 4-times before collecting the bottom EV-containing DEX-phase. To perform the PEG-phase washes, a large phase system was prepared by directly dissolving PEG and DEX in 40 mL DPBS utilising the same composition and method as previously applied to isolate EVs. The top PEG-phase of this large phase system was then collected and used to perform the repeat washes.

### Protein quantification

Protein was quantified utilising a Pierce BCA Protein Assay (Thermo-Fisher Scientific) according to the manufacturer’s instructions (Pierce, Rockford, IL). The absorbance was measured at 562 nm using a Varioskan Flash 4.00.53 plate reader with SkanIt Software 2.4.5 RE.

### Nanoparticle tracking analysis

Particle size and concentration was measured utilising Nanoparticle Tracking Analysis (NTA). EV fractions were diluted (1:70) in DPBS before injecting into the sample chamber using an automated syringe pump set to capture 5x measurements at 30-s long per run. Measurements were performed using the Nanosight LM10 instrument (Malvern Instruments Ltd) with a 488 nm laser. The software used for data capture and analysis was NTA 3.2 Dev Build 3.2.16. Temperature was kept at a constant of 22℃.

### Western blot analysis

EV containing fractions were loaded at a concentration of 5 µg and separation performed by electrophoresis on precast 4%–15% Mini-PROTEAN TGX gels (Bio-Rad). Proteins were transferred onto polyvinylidene difluoride (PVDF) membranes (Invitrogen), washed with 1x tris-buffered saline, 0.1% tween (TBST) and blocked in EveryBlot Blocking Buffer (Bio-Rad) for 5-min at room temperature. The membranes were then washed with TBST and incubated in Every Blot Blocking buffer containing primary antibody (see Table 1 for all antibody conditions) overnight at 4℃. Following overnight incubation, membranes were washed with TBST and incubated with horseradish peroxidase-conjugated (HRP) secondary antibodies in Every Blot Blocking buffer for 1-h at room temperature. The membranes were then washed in TBST, followed by incubation for 5-min in the dark with enhanced chemiluminescence (ECL) reagent (Bio-Rad). Detection of proteins was visualised with chemiluminescence using the ChemiDoc™ XRS+ Imaging System and analysis software Image Lab (Bio-Rad). Complete protein profiles were also obtained by staining the gels pre-transfer utilising Gelcode™ Blue Safe Protein Stain (Thermo-Fisher Scientific) overnight before imaging using the ChemiDoc™ XRS+ Imaging System and analysis software Image Lab.

**Table 1.** Western blot antibody and sample loading conditions.

Target Protein	Primary Antibody	Secondary Antibody
Alix	Mouse monoclonal 1:1000 Santa Cruz	Anti-Mouse 1:3000
Annexin A2	Rabbit polyclonal 1:2000 Abcam	Anti-Rabbit 1:3000
CD9	Rabbit polyclonal 1:1000 Abcam	Anti-Rabbit 1:2000
CD63	Rabbit polyclonal 1:1000 Abcam	Anti-Rabbit 1:2000
Calnexin	Rabbit Polyclonal 1:1000 Abcam	Anti-Rabbit 1:3000

### ExoELISA

The presence of tetraspanin markers CD63 and CD81 were quantified using the ExoELISA-ULTRA Complete Kit (System Biosciences) according to the manufacturer’s instructions. EV containing fractions were loaded at a protein concentration of 5 µg. The absorbance was measured at 450 nm using a Varioskan Flash 4.00.53 plate reader with SkanIt Software.

### Nano-flow cytometry

The presence of tetraspanin markers CD9, CD81 and CD63 were quantified by Nano-Flow Cytometry (NanoFCM) utilising a NanoAnalyzer U30 instrument (NanoFCM Inc.) with dual 488/640 nm lasers. EV containing fractions were diluted to a concentration of 1 × 10^10^ particles/mL in DPBS (Gibco, 10x diluted in HPLC grade water) and 9 µL of diluted EV containing fraction mixed with 1 µL of conjugated antibody (see Table 2 for antibody conditions). Samples were then incubated for 30-min at room temperature. Post-incubation, EV samples were diluted to a concentration of 1 × 10^8^–1 × 10^9^ particles/mL in DPBS for immediate phenotypic analysis. Data processing was performed using NanoFCM Professional Suite v1.8 software.

**Table 2.** Nano-flow cytometry antibody conditions.

Tetraspanin	Antibody conjugation	Supplier details	Antibody dilution
CD9	FITC-Conjugated Anti-Mouse	Clone EM-04 Abcam	1:200
CD63	APC-Conjugated Anti-Mouse	Clone NVG-2 Biolegend	1:100
CD81	APC-Conjugated Anti-Mouse	Clone EAT-2 Biolegend	1:100

### Transmission electron microscopy

EV containing fractions were prepared on continuous carbon (Cu 300 mesh TEM grids) fixed using 3% glutaraldehyde. Samples were then washed with milli Q water before negative staining with 1% uranyl acetate. Images were taken using a FEI Techni G2 12 Biotwin TEM, operating at 100 kV with a Gatan SIS Megaview iV camera.

### Survey: Data collection and analysis

Data was collated from 66 complete responses to **Q10** of a survey (Supplemental Materials, S1) looking at parameters governing implementation of EV isolation methods. The majority of respondents were from an academic background (81%). Eighteen percent of these respondents identified as having a therapeutic focus to their EV research. The survey was performed utilising Qualtrics survey software. It was opened November 2020 and closed February 2021. Ethical approval was granted by Loughborough University ethics committee (Reference 2020-2478-2026). Respondents were anonymised and informed consent obtained prior to participation. To meet the inclusion criteria respondents current research had to involve EVs, including personal experience of isolating EVs. Respondents were from varying research backgrounds and experience levels. Responses were given using a 1–5 ranking system as specified per parameter. Outputs were plotted as the mode. Multiple answers were given where applicable.

### Statistical analysis

Statistical analysis was performed using a one-way ANOVA followed by Tukey’s post hoc test with IBM SPSS software (IBM Analytics, version 24). A *p* value of 0.05 or less was considered significant, *p ⩽ 0.05, **p ⩽ 0.01,***p *⩽* 0.001. Graphs were created using software Origin 2020. Radar charts were created in Microsoft Excel 365.

## Results

### EV profiling

**Figure 2.**
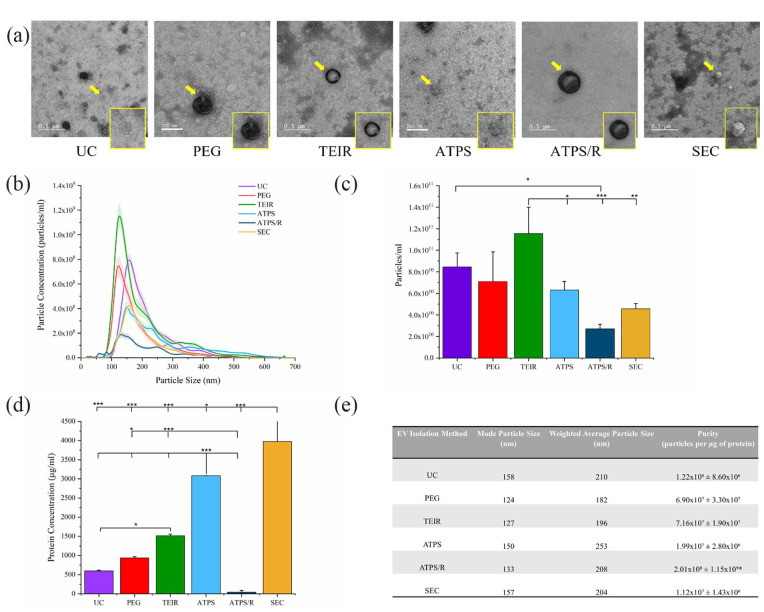
Profiling of EVs isolated by varying methods. EVs isolated from conditioned medium by UC, PEG precipitation, TEIR, ATPS, ATPS/R and SEC (combined fractions 2–10). (a) TEM images, (b) NTA particle size distribution, (c) NTA particles per ml and (d) BCA assay surface protein quantification. (e) NTA weighted average particle size, mode particle size and sample purity as a measure of PtP ratio. Data expressed as mean ± SD (*n* = 3), *p ⩽ 0.05, **p ⩽ 0.01 and ***p ⩽ 0.001.

Transmission electron microscopy (TEM) images confirmed the presence of EVs when isolating with all methods (Figure 2(a)). However, the presence of EVs was shown to be much lower for all precipitation-based methods (PEG, TEIR, ATPS an ATPS/R). UC TEM images showed the most abundant presence of EV material with visualisation of the characteristic EV lipid bilayer. The presence of electron dense background material was most evident for TEIR and SEC. NTA results displayed particle sizes within the typical range for EVs (30–200 nm), with the largest mode particle diameter observed for UC (158 nm), SEC (157 nm) and ATPS (150 nm) isolations (Figure 2(b)). The highest particle concentration was obtained with TEIR (1.15 × 10^11^ ± 2.44 × 10^10^ per mL) followed by UC (8.46 × 10^10^ ± 1.29 × 10^10^ per mL), PEG precipitation (7.11 × 10^10^ ± 2.74 × 10^10^ per mL), ATPS (6.31 × 10^10^ ± 7.89 × 10^9^ per mL), SEC (4.57 × 10^10^ ± 4.82 × 10^9^ per mL) and ATPS/R (2.71 × 10^10^ ± 4.09 × 10^9^ per mL) (Figure 2(c)). Significant differences in particle concentration were seen between TEIR and ATPS (*p* ⩽ 0.05), ATPS/R (*p* ⩽ 0.001) and SEC (*p* ⩽ 0.01), as well as between UC and ATPS/R (*p* ⩽ 0.05). Protein quantification (Figure 2(d)) revealed SEC to have the highest protein concentration (3974 ± 722.9 µg/mL) followed by ATPS (3081 ± 2.9 µg/mL), TEIR (1514 ± 43.7 µg/mL), PEG precipitation (938 ± 32.0 µg/mL), UC (601 ± 16.7 µg/mL) and ATPS/R (42 ± 46.2 µg/mL) with the lowest. Significant differences (*p* ⩽ 0.001) in protein concentration were seen when comparing SEC and ATPS to all other methods as well as ATPS/R to TEIR. In addition, significant differences (*p* ⩽ 0.05) were seen between SEC and ATPS, ATPS/R and PEG and UC and TEIR. Data outcomes above were used to calculate particle to protein ratio (PtP) as a basic measure of sample purity routinely applied across the EV field (Figure 2(e)). This measurement utilises the suggested principal that the presence of protein is a source of contamination and the presence of particles represents EVs. However, it should be noted that while frequently applied within the field, this measurement is not specific and does not take into account factors such as luminal proteins that may become released following EV lysis that could result from storage at −80°C or artefacts introduced by less specific isolation methods (e.g. precipitation). Based solely on PtP ratio, ATPS/R was found to have comparatively high levels of purity, with a significantly higher PtP ratio (*p* ⩽ 0.05) relative to all other methods except UC. SEC was indicated to have the lowest purity based on this calculation.

### EV characterisation

**Figure 3.**
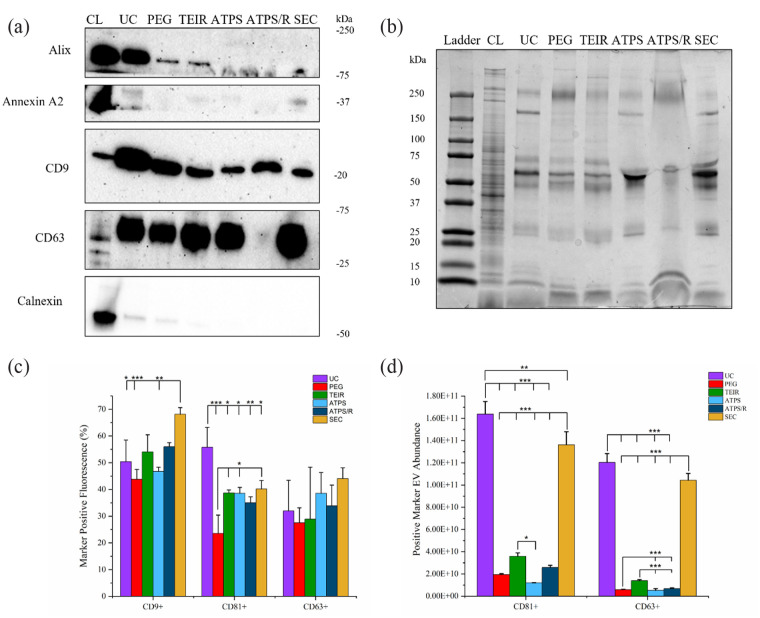
Characterisation of EVs isolated by varying methods. EVs isolated from conditioned medium by UC, PEG precipitation, TEIR, ATPS, ATPS/R and SEC (combined fractions 2–10). (a) Western blot analysis, cell lysate (CL) was obtained from C2C12 cells under culture conditions as stated. (b) Complete protein profile obtained using Gelcode™ Blue Safe Protein Stain. (c) NanoFCM CD9, CD81 and CD63 tetraspanin marker quantification. (d) ExoELISA CD81 and CD63 tetraspanin marker quantification. Data expressed as mean ± SD (*n* = 3), *p ⩽ 0.05, **p ⩽ 0.01 and ***p ⩽ 0.001.

Western blots (WB) were performed to qualitatively analyse the presence of common EV markers: Alix, annexin A2, CD9 and CD63 (Figure 3(a)). UC displayed strong bands for Alix, CD9 and CD63. PEG precipitation and TEIR also displayed strong bands for CD9 and CD63 and a visible band for Alix. ATPS and SEC displayed strong bands for CD9 and CD63 and ATPS/R only displayed a strong band for CD9. Annexin A2 was observed with only a faint band for all EV preparations. Calnexin was included as a negative marker (endoplasmic reticulum presence) and was shown to be strongly present in cell lysate (CL), as well as displaying weak bands for UC and PEG. The complete protein-stained gel (Figure 3(b)) indicated distortion of the gel with loading of EV fractions isolated by ATPS and ATPS/R. This is suggested to have negatively impacted the WB results, with major distortion indicated at the site of the CD63 band for ATPS/R which could not be identified in the WB results. Similar banding profiles were observed for EV preparations isolated using all other methods.

Quantitative validation of the presence of tetraspanin markers (CD9, CD63 and CD81) was assessed by NanoFCM (Figure 3(c)). Data showed that SEC had the greatest abundance of CD9℃ particles, which was significantly upregulated when compared to UC (*p* ⩽ 0.05), PEG precipitation (*p* ⩽ 0.001) and ATPS (*p* ⩽ 0.01). UC preparations demonstrated a significantly elevated presence of CD81^+^ particles when compared to all other isolation methods (PEG precipitation: *p* ⩽ 0.001; ATPS/R: *p* ⩽ 0.01; TEIR, ATPS, SEC: *p* ⩽ 0.05). PEG precipitation resulted in a significantly (*p* ⩽ 0.05) reduced presence of CD81^+^ particles when compared to all other isolation methods. Lastly, CD63℃ particles were most abundant when isolating by SEC, followed by ATPS, ATPS/R, UC, TIER and PEG precipitation. However, no significant differences were observed between isolation methods. ExoELISAs were utilised to further validate the presence of CD81℃ and CD63℃ particles (Figure 3(d)). The data indicated more pronounced differences when compared with previous NanoFCM outcomes, showing CD81^+^ and CD63℃ particles to be most abundant in UC and SEC preparations, with significant upregulation observed when compared with all other isolation methods (*p* ⩽ 0.001). CD81^+^ particles were also significantly (*p* ⩽ 0.01) upregulated in UC preparations when compared with SEC.

### Normalised quantitative analysis outputs

**Figure 4.**
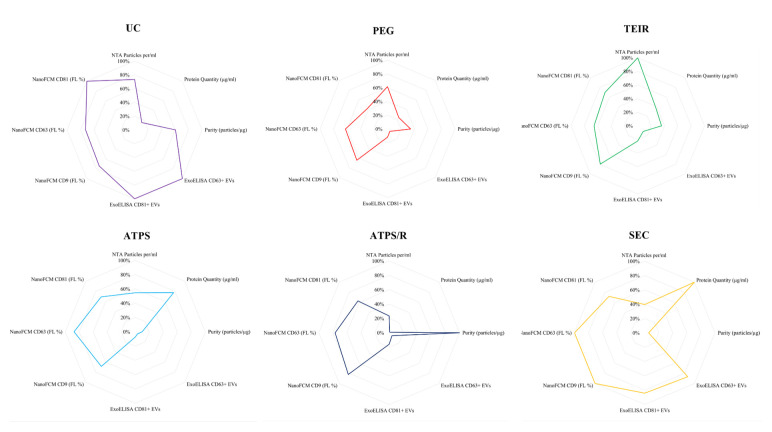
Normalised quantitative analysis outputs. Quantitative analysis outputs were normalised to a percentage of the method with the highest output. Methods include UC, PEG, TEIR, ATPS, ATPS/R and SEC. Analysis outputs as follows: NTA, protein quantity, purity, ExoELISA and NanoFCM marker quantification.

To understand the true scale of variability between EV isolation methods, we normalised all quantitative data to a percentage of the highest output and plotted radar charts for each method (Figure 4). Normalised data, when observing particle concentration, showed percentage differences to the highest output TEIR ranging from a minimum of 27% (UC), followed by 38% (PEG precipitation), 45% (ATPS), 60% (SEC) and a maximum of 77% (ATPS/R). Protein concentration showed a minimum percentage difference of 22% (ATPS) to the highest output (SEC), followed by 62% (TEIR), 76% (PEG), 85% (UC) and a maximum of 99% with ATPS/R. Purity, as a ratio of PtP, suggested a minimum percentage difference of 39% (UC) to the highest output ATPS/R, followed by 64% (TEIR), 66% (PEG), 90% (ATPS) and a maximum of 94% with SEC. When looking at marker presence we saw that when analysing by ExoELISA SEC and UC had consistently higher marker presence with a percentage difference between these two methods of 13% (CD63) and 16% (CD81). In comparison to larger percentage differences of 88%–96% to all other isolation methods. However, for the NanoFCM results we see a smaller percentage difference between all methods of 18%–38% across all markers. In addition, marker presence profiles for each isolation method were not consistent across analysis outputs.

### Survey results: ranking parameters governing method implementation

**Figure 5.**
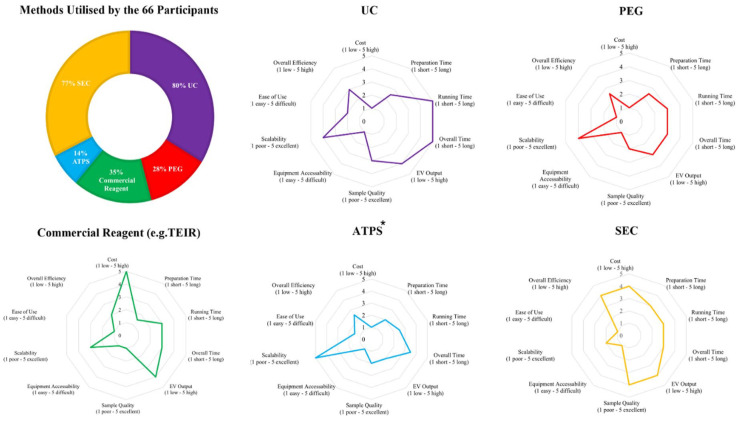
Survey results. Sixty-six complete responses from an internationally distributed survey looking at implementation parameters of EV isolation methods. Methods include UC, PEG precipitation, commercial reagents, ATPS and SEC. The percentage of respondents with experimental experience of each isolation method (all applicable selected) is displayed in the pie chart. Methods were ranked 1–5 (specifications on charts) for the following parameters: cost, preparation time, running time, overall time, EV output, sample quality, equipment accessibility, scalability, ease of use and overall efficiency. *ATPS data is based on 2 complete responses, remaining respondents who selected ATPS either did not select an answer or selected N/A.

In order for EVs to be applied therapeutically, it is important that isolation methods meet the requirements of the user within their working environment. With this in mind, a survey was distributed to understand how each isolation method is perceived by researchers across disciplines working with EVs (Figure 5). The 66 respondents of the survey indicated direct personal experience in application of UC (80%), SEC (77%), commercial reagents (35%), PEG precipitation (28%) and ATPS (14%). When evaluating cost, commercial reagents were ranked as most expensive (5) followed by SEC (4). While UC, PEG precipitation and ATPS were all ranked as low-cost methods (1). Preparation time was reported to be longest for SEC (3) followed by UC (2.5), PEG precipitation (2.5), ATPS (2) and commercial reagents (1.5). Running time was considered to be longest for UC (5) followed by PEG precipitation (3), SEC (3), commercial reagents (3) and ATPS (2.5). As a result, overall time was ranked longest for UC (5) followed by ATPS (3.5), PEG precipitation (3), commercial reagents (3) and SEC (3). EV output was considered to be highest for SEC (4), UC (4) and commercial reagents (4) followed by PEG precipitation (3) and ATPS (2). When looking at sample purity SEC was selected as most optimal (4) followed by UC (3), PEG precipitation (2), ATPS (2), and commercial reagents (1). All methods were deemed to be easily accessible in terms of equipment availability (1). The most scalable method was suggested to be ATPS (5) followed by PEG precipitation (4), UC (4), commercial reagents (3) and SEC (2). All methods were also indicated as easy to utilise, with PEG precipitation, commercial reagents and SEC considered to be the simplest (1), followed by ATPS (1.5) and UC (2). Lastly, overall efficiency was ranked highest for SEC (4) followed by UC (3), PEG precipitation (2.5), ATPS (2.5) and commercial reagents.

## Discussion

The recognised roles of EVs in diverse physiological and pathophysiological processes has generated increased interest in their prospective therapeutic and diagnostic applications.^2^ This interest has been further driven by the potential for EVs to overcome long-term challenges associated with cell-based therapies such as increased safety profiles and improved quality control, with the potential for off-the-shelf applications.^12,13^ However, translation is currently hindered by the inability to recover high yields of pure EVs. In addition to the availability of multiple diverse and often specialised EV isolation methods resulting in differential EV recovery, often limiting our ability to compare between studies and further hindering translation.^42^ Therefore, this study sought to compare a range of readily available and widely applied EV isolation methods, to determine their impact on EV purity and yield. Outcomes are presented in combination with the results of a survey that sought to evaluate additional pragmatic and logistical considerations related to the application of these methods in a standard laboratory setting. The resulting data allowed us to evaluate the overall feasibility in applying each of these isolation methods in therapeutically aligned research studies.

When comparing fundamental EV profiles between isolation methods, we observed variations in protein and particle concentration, purity and EV marker presence. Furthermore, these outcomes were highly dependent on the degree of specificity of the analysis applied, with total particle counts and routinely applied measures of purity repeatedly not aligning with the quantification of known EV surface markers such as tetraspanins. Particle concentration (Figure 2(c)) was found to be highest with TEIR (1.15E+11 per mL) and lowest with ATPS/R (2.71E+10 per mL). The application of such commercial reagents was also reported by respondents to be relatively time-efficient, easy to use and accessible (Figure 5). However, it should be noted that particles identified using NTA or other non-specific methods do not solely represent EVs, with the inclusion of co-isolated aggregated proteins and lipoproteins impacting the accurate interpretation of resulting data. This is particularly true for precipitation reagents such as TEIR, with the presence of such contaminants likely accounting for the higher particle numbers observed.^29^ Furthermore, commercially available reagents are costly, which was highlighted by respondents in the survey (Figure 5). In addition, the employment of these methods is dependent on continued availability of the product, potentially impacting long term scalable application. To more accurately determine the purity of our EV preparations we calculated the PtP ratio (Figure 2(e)). PtP or protein to RNA (PtR) are commonly applied measures of purity across EV studies.^43,44^ When applying PtP ratio, our results indicated ATPS/R (2.01E+08 particles per µg protein) exhibited the highest purity followed by UC (1.22E+08 particles per µg protein). These outcomes aligned with reports in the literature utilising ATPS/R for enhanced protein removal.^33,45^ Additionally, based on PtP ratio, SEC was found to recover EV preparations with the lowest overall purity (1.12E+07 particles per µg protein), with normalised data showing a percentage difference of 94% to the highest purity method (ATPS/R). However, when quantifying EVs based on the presence of tetraspanin surface markers using NanoFCM and ExoELISA (Figure 3(c) and (d)) we observed contrasting outcomes, with normalised data (Figure 4) indicating the highest recovery of tetraspanin-positive particles when applying SEC and UC. However, western blot results (Figure 3(a)) revealed that UC isolation did result in a noticeable calnexin band, which was not observed for SEC isolations and is indicative of endoplasmic reticulum contamination within the resulting EV preparation. The disparity between non-specific yet routinely applied measures of EV recovery with tetraspanin-positive particles highlights limitations when utilising particle counts and PtP as primary indicators of sample purity. PtP ratios cannot distinguish EVs from protein and RNA complexes or more recently identified subgroups such as exomeres^46^ and supermeres^47^ that may have distinct therapeutic or antagonistic effects. This has considerable implications for how we define therapeutic EV concentrations, with the majority of studies to date either defining dose by particle or protein concentration.^48,49^ Based on the findings from this study, these measurements may not accurately correlate with the presence of tetraspanin positive particles. Such variation in the evaluation of sample purity will impact our ability to determine therapeutic potency and introduce variation between studies utilising different isolation methods. Consequently, we identify the need to move towards the quantification of defined EV markers (e.g. tetraspanins) in a given preparation to improve reproducibility and better distinguish the therapeutic effects of EVs from other secreted factors.

The greater abundance of EVs observed when isolating by SEC and UC (based on tetraspanin marker presence), aligns with responses collected from our accompanying survey, which reported SEC and UC to be the most efficient methods overall (Figure 5). However, it should be noted that outcomes from this survey were based on the opinions of 66 respondents, with UC and SEC representing the most frequently applied methods. UC is widely compatible with all downstream analysis methods and was reported by respondents in our survey as being relatively accessible and cost efficient (Figure 5). The widespread application of UC, likely as a result of these factors, is further highlighted by its reported application in therapeutically focused clinical trials.^50^ However, as shown in both ours and other studies, low purity of EV preparations generated by UC could present downstream challenges in the translation of EV therapies.^51^ A major advantage of SEC over UC is its ability to selectively elute soluble proteins and lipoproteins in early fractions, aiding enhanced purification.^40^ To date, several studies have reported the use of SEC when working with complex biofluids for diagnostic biomarker applications^52–54^ to assist the removal of source-specific contaminants such as Tamm-Horesfall protein in urine^55^ or albumin in blood.^56^ Additionally, SEC has the potential to be applied downstream in therapeutic studies to enhance sample purification. However, it should be considered that increasing sample purity, although advantageous from a regulatory perspective, can sometimes have a negative impact on therapeutic efficacy. This has been best evidenced by Wolf et al. who observed a complete abrogation in angiogenic activity when purifying therapy-grade human placental-expanded stromal cell EVs using SEC or UC, due to the removal of surface bound material comprising the EV corona.^57^ Consequently, a compromise between sample purity and therapeutic efficacy may need to be taken into account when implementing regulatory measures for the advancement of EV therapeutics. Furthermore, when utilising SEC, a number of factors can impact EV recovery such as the cell type, culture system and the SEC protocol applied (e.g. commercially available or in-house produced columns, semi-automated or manual fraction collection).^17,39,58^ Therefore, the selection of EV containing fractions will likely need to be optimised based on these variables, which can be a time consuming process. This is also true for UC, where factors such as rotor type, g-force and centrifugation time have been shown to impact EV recovery.^59^ Notably, SEC was reported by respondents as being limited in scalability (Figure 5). This aligns with the observed need, exhibited in this study and others, too often combine SEC with UF when isolating from large volumes to concentrate samples pre-and/or post-isolation.^60,61^ However, these devices are costly and have also been shown to alter EV recovery dependent on the molecular weight cut off and membrane selected.^62^ Additionally, it should be noted that although UC was reported by respondents to be comparably scalable (Figure 5), this is likely based on the majority of responses being recorded by individuals working within a standard academic laboratory setting, with limitations in scalability anticipated when attempting to apply this method to process large volumes at an industrial scale. This limitation in scalability may potentially restrict the employment of these methods in the scale up to manufacturing of EV therapies.

Lastly, we will comment on the compatibility of EV isolation methods with downstream analytical techniques. This is perhaps best evidenced by the observed distortion of our protein gels for western blot when isolating by ATPS/R (final EV preparation in DEX) (Figure 3(b)). In this example we detected masking of CD63 on the protein gel. However, this marker could be clearly identified when using both NanoFCM and ExoELISA. In addition, relatively few EVs were observed in TEM images for ATPS/R preparations, as well as for other EV preparations isolated using a precipitation-based method (PEG, TEIR and ATPS). All of these isolation methods contain additional viscous reagents that have been shown to have a residual presence in EV preparations that can interfere with downstream analysis methods such as western blot,^34^ TEM^25^ and mass spectrometry.^26^ Our study also emphasised disparity between results obtained using ExoELISA and NanoFCM for EV preparations isolated using precipitation-based methods. This is perhaps explained by the fact that ExoELISA assays rely on absorbance outputs, which could potentially be impacted by the viscosity of residual precipitation reagents, whereas NanoFCM provides single particle analysis based on light scattering and fluorescent antibody detection. Consequently, the presence of any residual precipitation reagent could also potentially introduce variations in the light-scattering properties of EVs. The addition of further cleanup steps by combining precipitation methods such as PEG with UC has shown potential, with positive results observed for therapeutic efficacy.^63^ However, the clinical safety of reagents such as PEG has been brought into question,^64^ while the immunogeniticy of DEX is largely unknown. Despite this, promising application of EVs isolated by PEG precipitation has been demonstrated clinically for the treatment of graft versus host disease.^65^ PEG is also incorporated in anti-cancer medications such as Doxil^21^ and the mRNA-based covid vaccinations.^22^ However, its application for the isolation of therapeutically potent EVs requires further evidence.

### Summary and conclusion

This study looked at the impact universally applied EV isolation methods have on purity and yield, as well as pragmatic considerations for their implementation in developing EV therapeutics. All protocols applied were able to isolate EV-enriched fractions. However, outputs were highly variable between isolation methods, with some methods being incompatible with common analysis methods such as western blotting. This variation in EV recovery has considerable implications for assessing biological functions between studies and could negatively impact EV dosing and therapeutic outcomes, thereby hindering clinical translation. Overall, our data highlighted that SEC and UC recovered EV-enriched fractions with the highest tetraspanin marker presence. We evidenced disparity between tetraspanin positive particle recovery and other routinely utilised measures of EV purity, highlighting a need to move towards the quantification of defined EV markers to aid reproducibility. In conclusion, isolation methods should be carefully selected to ensure compatibility with downstream analyses and applications. Based on our findings it is likely that introducing variations in an EV isolation protocol as a therapeutic study moves from bench to bedside could lead to changes in the EV preparation that could impact therapeutic potency. Further investigation is required in this area to ensure consistency throughout the development pipeline.

## Supplemental Material

sj-docx-1-tej-10.1177_20417314231174609 – Supplemental material for Comparison of extracellular vesicle isolation processes for therapeutic applications Click here for additional data file. Supplemental material, sj-docx-1-tej-10.1177_20417314231174609 for Comparison of extracellular vesicle isolation processes for therapeutic applications by Soraya Williams, Maria Fernandez-Rhodes, Alice Law, Ben Peacock, Mark P. Lewis and Owen G. Davies in Journal of Tissue Engineering
