# Supplementary material for: Comparison of extracellular vesicle isolation processes for therapeutic applications
Source: J Tissue Eng. 2023 May 23;14:20417314231174609. doi: 10.1177/20417314231174609 (PMC10214056; doi:10.1177/20417314231174609)

Supplementary Information

EV Researchers Experience of EV Isolation Methods Online Questionnaire

Start of Block: Block 1

**EV Researchers Experience of EV Isolation Methods Online Questionnaire**

**PARTICIPANT INFORMATION**

**What is the purpose of the questionnaire?**

A current limiting factor of EV research is the suboptimal EV isolation methods. My current PhD research compares five of the multitude of EV isolation methods, in order to understand their advantages and disadvantages for varied applications and research settings. The purpose of this questionnaire is to collate personal experience and opinions of EV isolation methods, from both new and established EV researchers, across the disciplines of EV research. The information collected will then be compared alongside my own findings, to give an up to date understanding of these applications and challenges of EV isolation methods within the wider EV community.

The survey consists of 11 short multiple-choice questions and should take around 4 minutes to complete.

**Are there any exclusion criteria?**

Your current research must involve Extracellular Vesicles

You must have laboratory experience of isolating Extracellular Vesicles

**Investigators Details:**

If you have any questions, please do not hesitate to contact the Principal Investigator, Soraya Williams *(s.williams3@lboro.ac.uk)*, or her PhD supervisor Dr Owen Davies *(O.G.Davies@lboro.ac.uk)* at Loughborough University, UK.

The questionnaire will complete once you have pressed the submit button on the final page.

Please answer the questions focusing on your own personal laboratory experiences.

End of Block: Block 1

Start of Block: Block 2

**ONLINE SURVEY INFORMED CONSENT LANDING PAGE**

**Welcome to the EV Researchers Experience of EV Isolation Methods Survey**

**Taking Part**

The purpose of this survey is to record your own personal experience and opinions of EV isolation methods.

This study is designed to further scientific knowledge and that all procedures have been approved by the Loughborough University Ethics Approvals (Human Participants) Sub-Committee.

Please remember to answer the questions honestly throughout the survey.

You are under no obligation to take part in the study, you have the right to withdraw from this study at any stage for any reason and will not be required to explain your reasons for withdrawing.

**Use of Information**

I understand that all the personal information I provide will be processed in accordance with data protection legislation on the public task basis and will be treated in strict confidence unless (under the statutory obligations of the agencies which the researchers are working with), it is judged that confidentiality will have to be breached for the safety of the participant or others or for audit by regulatory authorities.

I understand that information I provide will be used for PhD research in the form of reports for research output purposes and scientific publication.

I agree that information I provide can be quoted anonymously in research outputs.

I agree to assign the copyright I hold in any materials related to this project to Soraya Williams.

I agree for the anonymised data I provide to be deposited in Qualtrics so that it can be made publicly available for future research at the end of the project.

I understand that cookies and personal data stored by your web browser are not used in this survey.

- **I voluntarily agree to take part in this study, for our records and to confirm your agreement with the above, please select**

*The answers to Q1-9 and 11 are being utilised in another study*


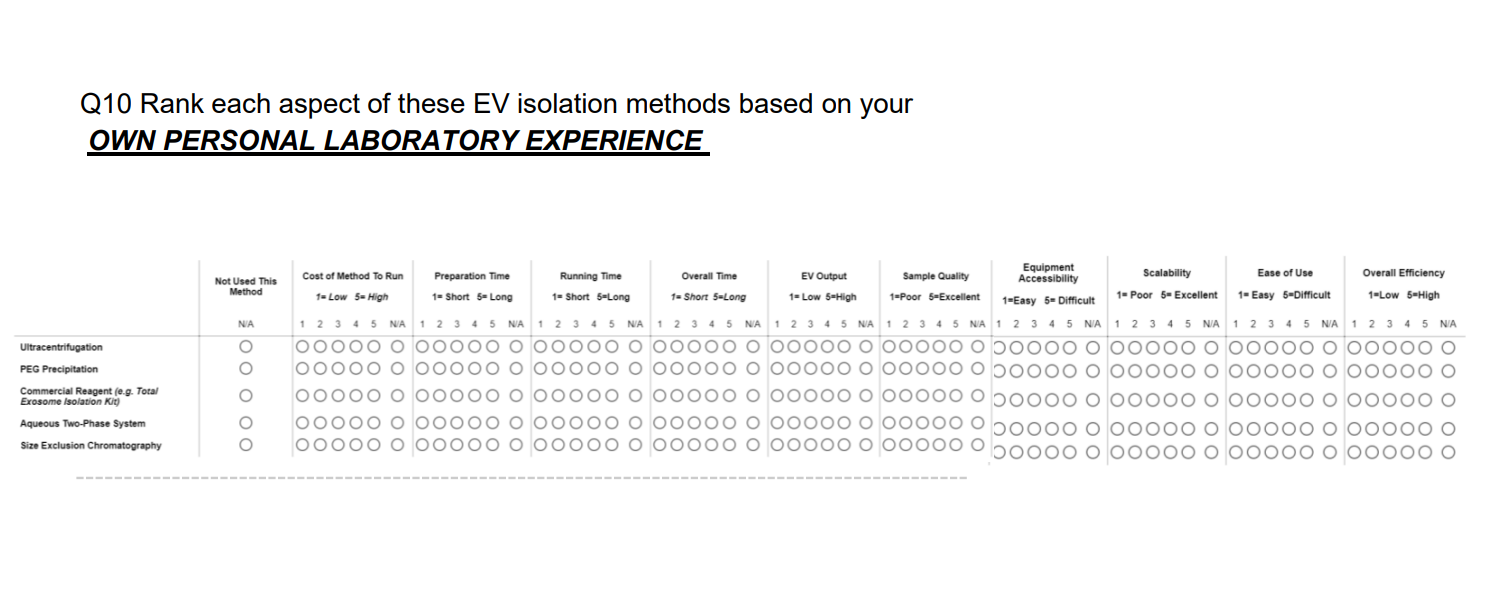

Supplement: sj-docx-1-tej-10.1177_20417314231174609 – Supplemental material for Comparison of extracellular vesicle isolation processes for therapeutic applications [file sj-docx-1-tej-10.1177_20417314231174609.docx]
